# Nurses’ Protests during the COVID-19 Pandemic: A Comparative International Analysis

**DOI:** 10.3390/nursrep14030146

**Published:** 2024-08-09

**Authors:** Davina Jacobi, Tobias Ide

**Affiliations:** 1 Discipline Area of Nursing, IUBH International University, 99084 Erfurt, Germany; 2 Center of Biosecurity and One Health, Murdoch University, Murdoch, WA 6150, Australia

**Keywords:** care worker, corona, health worker, nurse, politics, protest

## Abstract

Nurses play key roles in dealing with pandemics yet are often conceived solely as “technical” experts without political agency. This study conducts the first global comparative analysis of COVID-19-related protests of nurses and other frontline health workers, with a focus on the first 18 months of the pandemic. We draw on quantitative and qualitative data on nurses’ protests and protest drivers. Results show that such protests were widespread: We identify 3515 events in 90 countries, with several regional hotspots existing. The most common reasons for protests were poor working conditions and insufficient workplace safety, followed by wider social issues like poverty and racism. For most of the time period under consideration, protests demanding access to vaccinations (a rarely explored phenomenon) were more widespread than anti-vaccination events. Protest frequency was highest in countries with high COVID-19-related mortality rates, high levels of human development, and strong social movements at the onset of the pandemic. Recognising the key role of nurses as political actors would help to improve health policies and to maintain a capable healthcare workforce, particularly during acute crises like pandemics.

## 1. Introduction

The COVID-19 pandemic posed a major challenge to health systems and human development. According to Mathieu et al. [1], at least seven million people had died from the SARS-CoV-2 virus by early May 2024, while many more have suffered intense symptoms, including long COVID [2]. Due to infections, lockdowns, and border closures, almost five years’ worth of human development progress were lost, with poor countries being particularly affected [3]. The pandemic was also heavily politicised. People around the world protested against COVID-19-related restrictions and even attacked health workers [4], while actors as diverse as governments, companies, and criminal cartels implemented measures to limit the spread of COVID-19 and to promote vaccinations [5].

Nurses and other health workers were on the forefront of those efforts. They screened for COVID-19, educated about measures to limit the spread of the disease, cared for infected patients, and rolled out vaccinations. Particularly in frontline roles, they faced significant risks during the COVID-19 response, including higher infection rates [6], stigmatisation and harassment [7], long work hours, unpaid overtime and burnout [8,9], increased levels of psychological stress [10,11], and physical violence [4].

Consequentially, nurses joined political struggles around the pandemic by publicly protesting against their situation. Examples include a sit-in by Algerian nurses in the town of Blida to raise awareness about insufficient equipment and supplies to deal with the surging number of COVID-19 patients (15 March 2020); public protests in Berlin, Germany, for better working conditions in the healthcare sector (20 May 2020); and public demands by nurses in Tacna, Peru, to gain better access to vaccinations (11 March 2021). Despite this, nurses are often considered unpolitical actors, valued for their technical expertise and dedication, and sometimes discussed as passive victims of bad policies [9]. Only occasionally, if at all, are nurses considered active participants in political processes. Our study challenges this bias by analysing protests by nurses and other frontline health workers worldwide in the first 18 months of the COVID-19 pandemic.

By doing so, we contribute to the small but growing literature on protest activities by health workers [12,13]. In contrast to other studies, we focus on manifest, public protests, rather than individual coping strategies (e.g., leaving the sector) or institutionalised bargaining (e.g., through unions) [8,14]. While studies on individual countries exist (e.g., [12,15]), we provide the first systematic, globally comparative analysis of health worker protests during the COVID-19 pandemic (the Health Worker Protest Project aimed to build and analyse a similar database, but was discontinued in late 2020 and is no longer publicly available [16]).

In sum, our study aims to provide a global comparative analysis of COVID-19-related protests of nurses and other frontline health workers during the first 18 months of the pandemic. In the remainder of the paper, we first introduce our data and methods (Section 2) before presenting (Section 3) and discussing our results (Section 4). The conclusions (Section 5) summarise key insights, potential shortcomings, and directions for future research.

## 2. Materials and Methods

### 2.1. Definitions and Data

The primary source of information for this study was the Ared Conflict Location and Event Data Project (ACLED). The project compiles and curates a global inventory of conflict events, ranging from protests and riots to battles and terrorist attacks. To do so, ACLEDs draws on a range of news media as well as in-country experts, and data are usually screened and coded by humans. The dataset is publicly available [17].

We followed ACLED’s definition of protests “as a public demonstration in which the participants do not engage in violence, though violence may be used against them. Events include individuals and groups who peacefully demonstrate against a political entity, government institution, policy, group, tradition, businesses or other private institutions” [18]. Protests are usually a result of two factors: grievances that motivate individuals to voice their opinions, and opportunities that allow protests to happen, such as a lack of state repression or the option to coordinate via social media [19].

This study focusses on protests by nurses and other frontline health workers during the first 18 months of the COVID-19 pandemic, from 1 March 2020 to 31 August 2021. Our analysis is global in nature but excludes Australia, Canada, New Zealand, and the Pacific Island states because ACLED only started to cover these from January 2022 onwards. Data were extracted from December 2021 to February 2022 (while ACLED raw data are published almost in real time, it takes several weeks until the data are cleaned and verified).

We employed a multi-stage strategy to identify protest events that fulfilled our inclusion criteria, that is, were related to COVID-19 and initiated by nurses or other frontline health workers. In a first step, we extracted all events that mentioned “corona” or “COVID” in the ACLED event description. Afterwards, we employed the following Boolean search string to further narrow down the results: “nurse OR health worker OR healthcare worker OR healthworker”. This resulted in a list of 4247 events. After removing duplicates and false positives manually, the final sample contained 3515 protests in 90 countries.

### 2.2. Data Analysis

The study presented here asks four key questions. First, what was the spatial distribution of protests, and which countries were particularly affected? Second, what were the stated reasons for protests? Third, how did protest patterns change over time? Fourth, which socio-economic conditions can explain higher protest occurrence?

The first and third questions can be answered based on descriptive statistics because ACLED contains the date and place (country) for each protest. To address the second research question, we identified eight (not mutually exclusive) reasons for protests. We developed these reasons inductively from the data, but also ensured they are in line with wider research on nurse protests [12,15] and protests around COVID-19 [20,21]. Both authors coded each event separately, and any differences were subsequently resolved through conversations. Table 1 provides an overview about the categories together with an example for each one.

To answer the fourth question, we identified five scope conditions that could shape the occurrence and frequency of protests at the country level. They include (1) the level of human development according to the 2019 Human Development Index (HDI) [22]; (2) the number of care workers per 1000 inhabitants in 2019 as an indicator for the quality of a health care system, based on data from the World Bank for 2019 [23]; and (3) the level of democracy, measured by the polity2 value assigned to a county by the Polity V Project for 2019 [24] (we transformed the polity2 scores into values from 0 to 20 for this purpose). In addition, we aimed to study whether the severity of the pandemic and the presence of countermeasures affect protest dynamics. This study hence also utilised data on the percentage of the population that received at least two shots of a COVID-19 vaccine and on the confirmed number of COVID-19-related deaths per million inhabitants as of 31 August 2021 (the last day covered by our analysis). Data came from the Our World in Data Coronavirus Pandemic Tracker [1]. Given the rather small N (*n* = 90) of our analysis, we ran descriptive statistics as well as simple bivariate correlations (Pearson’s R and *p*-value).

## 3. Results

### 3.1. What Was the Spatial Distribution of Protests, and Which Countries Were Particularly Affected?

**Figure 1:**
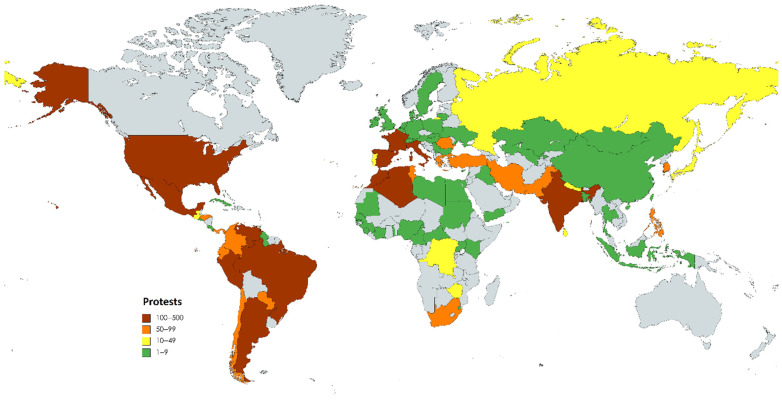
Distribution of protests globally.

Overall, we identified 3515 protest events in 90 countries. Figure 1 visualises the global distribution of protests, with 12 countries having experienced more than 100 events: USA (500), France (319), Mexico (270), Argentina (246), Venezuela (204), Italy (194), Spain (172), Peru (141), Morocco (140), India (140), Algeria (106), and Brazil (104). One should note that in some countries, media censorship, lockdowns, and poor transport infrastructure are likely to cause an underreporting of protest activities. This might explain why we did not find any protests in Afghanistan, Ethiopia, Somalia, Syria, or South Sudan.

**Figure 2:**
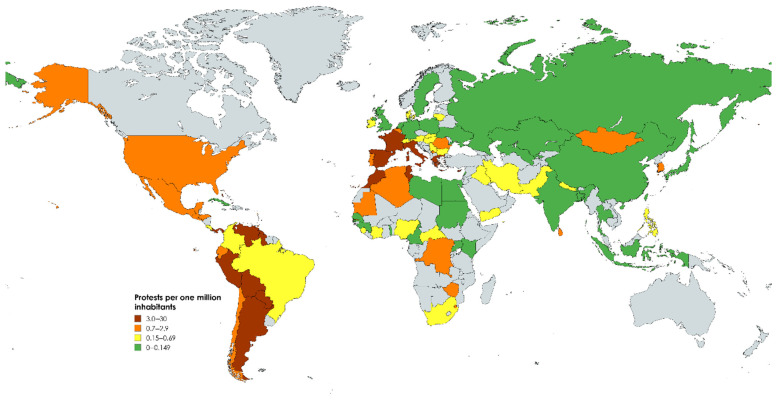
Protest per one million inhabitants.

Reporting the absolute number of protests can be misleading because it involves comparing population-rich countries with very small countries. Figure 2 hence displays the number of protests per one million inhabitants. While some protest hotspots remained identical (e.g., Argentina, France, Italy, Peru, Spain, Tunisia, USA, Venezuela), we found the highest protest frequency in small states like San Marino (29.5) and the Bahamas (12.7), while large countries like Brazil (0.49) and particularly India (0.1) moved down the list (see the Supplementary Materials for the full dataset).

### 3.2. What Were the Stated Reasons for Protests?

**Figure 3:**
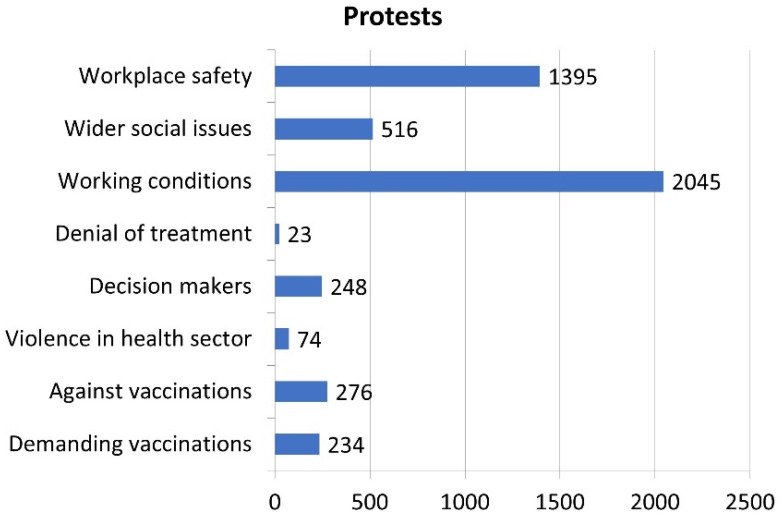
Reasons for protests (*n* = 4811 due to several protests having multiple reasons).

Figure 3 provides a summary of the reasons for protests, based on our coding of all 3515 events discussed in Section 2 (see Table 1 for an illustrative example of each event). Working conditions were the most important driver of protests (42.5%), followed by workplace safety (29%). All other reasons played only a minor role, in particular, protests against health sector violence (1.5%) and against treating COVID-19-positive patients (0.5%). Altruistic motives in the form of wider social issues were the third most common reasons for protests (10.7%), while protests demanding (5.7%) and opposing (4.9%) vaccinations had a similar frequency.

**Figure 4:**
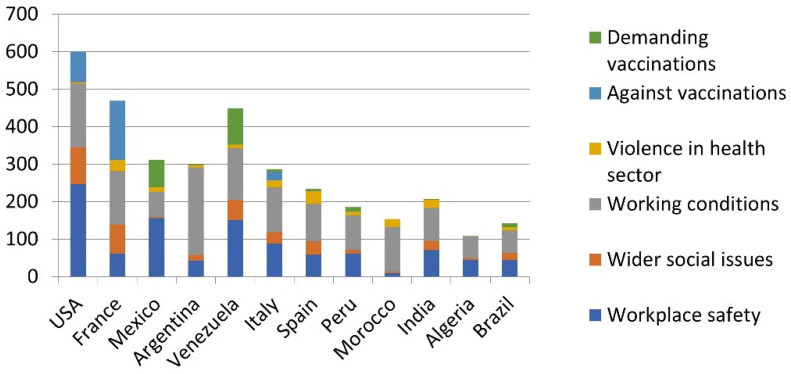
Protests reasons by country (with a focus on the six most common causes).

Figure 4 disaggregates the protest reasons for the 12 countries with more than 100 events. Working conditions and (with the exception of Morocco) workplace safety were the by far dominant protest topics in all countries. France (16.4%), the USA (16.3%), and Venezuela (11.8%) saw unusually large numbers of protests related to wider social issues, such as vaccination and lockdown policies. In line with our expectations, poor countries that suffered from significant vaccination scarcities like Mexico (23.7%) and Venezuela (21.6%) saw the largest number of protests about a lack of vaccination.

### 3.3. How Did Protest Patterns Change over Time?

**Figure 5:**
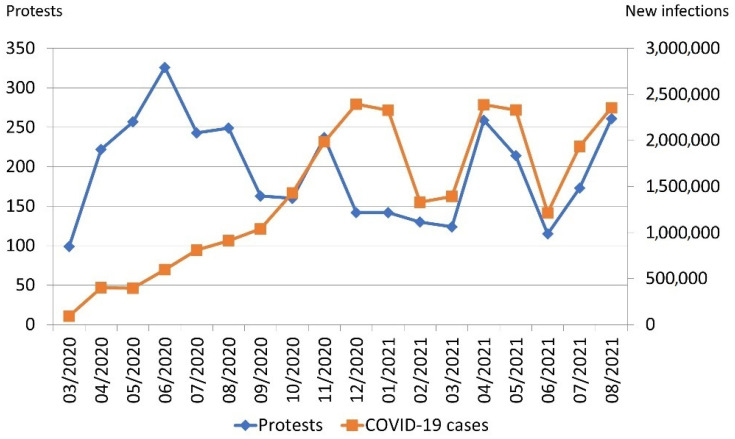
Protests (blue line) and COVID-19 infections (red line) per month.

In the first six months of the pandemic, large numbers of protests by health workers occurred (despite low case numbers). There are several reasons behind this: The virus was new, so governments were not well prepared (e.g., in terms of stocking equipment) and insecurity among health workers about the (long-term) health impacts of COVID-19 was high (see Section 4 for further discussion). In line with this, workplace safety was the most frequent reason for protests from March to May 2020. From October, the number of protests closely followed the number of new infections per month, with protest spikes in April 2021 and August 2021 (see Figure 5).

There were several notable patterns when comparing the reasons for protests over time (full data in the Supplementary Materials). For most of the period studied here, workplace safety (March–May 2020) and especially working conditions (June 2020–June 2021) were the most important reasons for protests. A total of 69.6% of all protests indicating a denial of treatment happened in the first six months of the pandemic, indicating that uncertainty among nurses declined with more information about the pandemic becoming available.

Public health worker demand for vaccination access start in December 2020 and clearly outweighed protests against vaccinations until June 2021 (217 vs. 19 events). However, this relationship reversed in July and August 2021 (26 protests demanding vaccinations and 255 protests opposing vaccinations). In those two months, resistance to vaccination was the most important reason behind health worker protests. The emergence of vaccination mandates, both for nurses and for participating in public life, drove this development. France, for instance, became the country with most COVID-19-related nurse protests in July and August 2021 (157 events). A total of 98.7% of these were directed against vaccinations and closely connected to President Emmanuel Macron’s introduction of a vaccination mandate for health workers as well as a health pass to access places like restaurants or museums [25].

### 3.4. Which Socio-Economic Conditions Can Explain Higher Protest Occurrence?

In a final analytical step, we assessed the socio-economic conditions that drove high protest occurrence by nurses and other frontline health workers (relative to a country’s population), based on the data introduced in Section 2. In other words, Which characteristics influence whether a country experiences a high or low number of protests (relative to its population)?

We expected a high number of care workers per capita, which is a common indicator of the quality of health systems, to reduce protest frequency. In well-funded (and hence well-staffed) health systems, nurses should have fewer incentives to protest during the pandemic, particularly because working conditions and workplace safety were the two main reasons for protests. In line with this, the correlation between protest occurrence and health workers per capita in a given country is negative. However, the correlation is only very weak (Pearson’s R = −0.0034) and far from statistically significant (*p* = 0.928).

Our second assumption was also not confirmed by the analysis. In theory, our dataset should contain more protests by nurses in democratic political systems. Autocratic states are likely to supress protests and, even if demonstrations occur, prevent them from being widely reported [26]. But the correlation between the polity2 value of a country and its protest frequency is weak (R = 0.1371) and not significant (*p* = 0.208).

The correlation between health worker protests and vaccination rates is positive (R = 0.2763) and significant at the 5% level (*p* = 0.043). The finding is not robust, however, but rather driven by the (11) countries with very low vaccination rates (≤10%). In many of those countries, protests are likely to be underreported due to political instability as well as weak and restricted media systems (e.g., Bangladesh, Democratic Republic of Congo, Pakistan). On average, countries with high vaccination rates (≥50%) even see slightly fewer protests (1.52 protests/million inhabitants) than countries with medium vaccination rates (25.1–50%, 1.81 protests) and low vaccination rates (10.1–25%, 1.91 protests).

**Figure 6:**
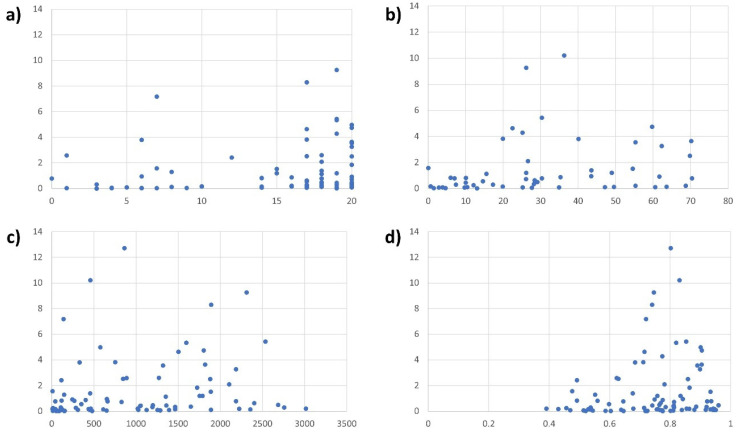
Scatterplots visualising the relationships between the number of nurse and health worker protests per one million people (y-axis) and the level of democracy (**a**), double vaccination rates (**b**), COVID-19-related deaths per one million inhabitants (**c**), and the level of human development (**d**).

By contrast, the number of COVID-19-related deaths is a positive (R = 0.3164), significant (at the 1% level, *p* = 0.002), and robust predictor of pandemic-related protests by nurses at the country level. Likewise, we find evidence that high human development levels are associated with more protests, even though the correlation is weaker (R = 0.1926) and only significant at the 10% level (*p* = 0.074). Figure 6 visualises several of the relevant correlations.

## 4. Discussion

When analysing the spatial distribution of COVID-19-related protests by nurses and other healthcare workers, four distinct regional clusters can be identified. First, in the USA, relatively high infection rates and a strong politicisation of the pandemic during the Trump presidency combined to trigger widespread protests. Second, South America and Mexico experienced a high frequency of protests, most likely due to the heavy impacts of the first two waves on the pandemic and rather weak healthcare systems [27]. When the pandemic struck, countries like Chile and Venezuela quickly saw large-scale social protests [27], hence facilitating the politicisation of COVID-19. A third protest hotspot was Southern Europe, particularly Greece, Italy, and Spain. These countries were struck by a heavy first COVID-19 wave and have a strong protest culture, especially in France [1,28]. Fourth, parts of North Africa and the Middle East experienced high levels of health worker protests. Here, it seems again that in countries that saw large-scale protests in 2019 and hence had well-organised social protest movements (Algeria, Morocco, Tunisia), health workers voiced their discontent more openly compared to, for example, Egypt [29].

This argument is further supported by the analysis of the reasons for protests. Probably the most political category of protests, namely, those related to wider social issues, was most prevalent in countries that had already experienced protest movements and political polarisation before the pandemic, such as France, the USA, and Venezuela (see also Figure 4). Protests against political decision-makers were also far from uncommon (5.2% of all events). Together with the overall high number of protests, this shows that nurses and health workers frequently acted as “political activists” ([13] p. 1), not just as technical experts or passive victims of health policies and violent offenders. While the anti-vaccination movement has received significant attention [30], researchers and practitioners should also study pro-vaccination protests. These were almost as frequent as anti-vaccination events in our sample and indicate strong bottom-up demand for evidence-based healthcare as well as for the associated tools. This would also connect well with existing work on the psychological impacts of COVID-19, which can be amplified by both a lack of vaccinations and vaccination mandates [11].

When analysing the temporal trends, it became clear that after an initial period of uncertainty, protest numbers closely followed infection numbers. Insufficient policies to contain infections and prepare health systems hence not only overburden the health system but also result in massive grievances among those most needed during the height of a pandemic: frontline health workers. This also illustrates that nurses can be significant agents of positive social and political change by voicing public demands for better health policies [13]. However, this is not always the case. Health worker protests in July and August 2021 voiced strong opposition to vaccination, despite strong scientific evidence for their benefits. However, such claims only represent a minority opinion. While France was the epicentre of anti-vaccination protests, for instance, 78% of all French had received two doses of vaccination by late August 2021 [1]. Further research on the impacts of nurses’ protests and other forms of political action could further nuance these insights [14].

Contrary to our expectations, the number of care workers per capita (used here as an indicator of the quality of a country’s healthcare systems) showed no significant correlation with protest occurrence. One reason for this could be that the positive effects of a high health worker density (strong healthcare systems reducing concerns about working conditions and workplace safety) is set off by a negative effect related to opportunities for protest mobilisation: In countries with many nurses and doctors, protests become more likely simply due to a higher number of relevant actors [19]. We also found no robust impact of the political system or of vaccination rates on protest frequency. The former finding could be due to the fact that during the pandemic, even many democratic governments imposed legal restrictions on protest activities [31].

Our analysis shows that COVID-19 deaths are the best predictor of strong protest activities by nurses and other frontline health workers. This is well in line with the temporal trends of new infections and protest numbers largely running in parallel to each other. A high number of COVID-19 deaths is also closely connected to the second main reason behind protests: workplace safety (the risk of becoming seriously unwell or dying from COVID-19), and it adds salience to all other causes of protests (except for anti-vaccination demonstrations), including to the main reason for protests: working conditions (e.g., via high workloads and high psychological stress) [10]. Contrary to our expectations, we found that high (rather than low) levels of human development were associated with more protests. Possible explanations for this include that in less developed economies, nurses are too scared to protest (because they could be laid off while unemployment rates are high), too busy with activities other than protesting to sustain their livelihoods, or disillusioned with the possibilities for improved working conditions and work safety given the limited resources available in poor countries [32].

One shortcoming of this analysis is the rather coarse resolution of the data, which are aggregated on the country level and either monthly or for the whole period under study. While in our sample, many protests can be linked to national-level decisions and developments, drivers of protests are also often local and display high degrees of temporal variation. On 3 December 2020, for example, nurses in Seoul gathered in front of the metropolitan government building to demand better COVID-19-related health provisions for homeless people. The protest was strongly driven by local initiatives and seasonal factors (the onset of winter). Disaggregating the data by lower-level administrative units and by months or even days is a promising direction for future research. Likewise, updating the data to include 2022 and 2023 would reveal insights into how the widespread availability of vaccines and eventually the depoliticization of the pandemic affected protest dynamics.

## 5. Conclusions

This study presented the first global analysis of COVID-19-related protests by nurses and other frontline health workers. Focussing on the first 18 months of the pandemic, we identified and coded 3515 protest events in 90 countries. This demonstrates that COVID-19-related protests by health worker are a global and significant phenomenon.

We went on to uncover the motives behind, temporal trends of, and socio-economic correlates of protests. The most common reasons for protests were working conditions and workplace safety. However, nurses also went to the streets to raise political issues that affected them less directly, hence demonstrating considerable altruism. Relevant reasons for protests include wider social issues (e.g., poverty, racism) or contested political figures. For almost the complete period under study, protests demanding access to vaccinations were more widespread than anti-vaccination demonstrations. After an initial phase of uncertainty, protest activity closely followed infection rates. In line with this, the number of COVID-19-related deaths was the strongest predictor of the number of protests a country experienced. Countries with high levels of human development (where relative job security enables political action, while available resources incentivise political action) and countries with large-scale social movements or political polarisation during the onset of the pandemic experienced the highest number of protests (relative to their population).

We have shown throughout this study that nurses are not only experts in the healthcare sector but also deeply political actors (rather than just passive bystanders or victims). If they carry their grievances and demands to the street, it can be a strong indicator of serious problems in the sector. Given their relevance in providing health services, particularly in times of a pandemic, politicians and decision-makers are well advised to listen and react to these demands. This is in line with Karen H. Morin and Diana Baptiste’s call to support protesting nurses who “take risks by voicing loudly issues threatening patient safety in healthcare organisations and in the larger public area” ([11] p. 1). Regular communication channels between frontline health workers and those making health-relevant decisions, including executives of healthcare institutions and political decision-makers, should be strengthened to facilitate exchanges before protests erupt.

## Figures and Tables

**Table 1:** Eight reasons for protests and examples, based on ACLED [17].

Reasons for Protest	Example
Protests for workplace safety (e.g., insufficient equipment, lack of personnel, improper training)	“On 31 August 2021, members of KCTU Incheon and Incheon Regional Solidarity picketed in front of Incheon Metropolitan City Hall. They urged the government to solve the lack of nursing personnel and provide a better working environment for health workers amid the coronavirus outbreak.”
Protests related to wider social issues (these are not job-specific, but concern wider health policies, social security, etc.)	“On 7 March 2020, nearly a dozen nurses, activists and community members protested outside the city hall in Baltimore (Maryland) demanding protection for the most vulnerable to the coronavirus disease. They urged the creation of a distribution food plan, the suspension of evictions, the distribution of school lunches to vulnerable children, the aid for homeless people in shelters and assistance for immigrants.”
Protests related to working conditions (predominantly related to payment, compensation, job security, and workload)	“On 3 July 2020, in Lima, a group of health workers of the Arzobispo Loayza National Hospital protested complaining they had not received the coronavirus bonus announced by the Government, as well as the payment for the additional hours they performed.”
Protests indicating denial of treatment (health workers refuse to treat COVID patients due to infection risks)	“On 16 April 2020, health workers in the regional hospital of Medenine protested to prevent the transfer of a patient from Djerba allegedly infected with coronavirus.”
Protests against decision-makers (directly criticising them, demanding them to take action, or calling for their removal from office)	“On 31 July 2020, in Tonala, Chiapas, health workers demonstrated to demand the release of a doctor arrested for alleged abuse of power and to demand the dismissal of the Chiapas Secretary of Health.”
Protests against violence in the health sector (voicing grievances about physical or verbal attacks against health workers)	“On 13 June 2020, health workers held a protest sit-in in Annaba, denouncing the aggression on one of their colleagues by a patient infected with coronavirus.”
Protest against vaccinations (opposing vaccination mandates and similar policies)	“On 17 July 2021, between 1000 and 1200 people, including yellow vest demonstrators and health workers, protested in Nancy against coronavirus-related measures such as a mandatory ‘health pass’ to access French cultural venues as well as compulsory vaccination for all health workers.”
Protests demanding vaccinations (health workers asking for access to vaccinations)	“On 21 May 2021, in the Palavecino municipality, Lara state, health workers staged a protest to demand the effective provision of the coronavirus vaccine.”

## Data Availability

All data used in this article are publicly available from the sources cited. Key data can be found in the Supplementary Materials.

## Supplementary Materials

The following supporting information can be downloaded at: https://www.mdpi.com/article/10.3390/nursrep14030146/s1, Supplementary dataset.

## Public Involvement Statement

No public involvement happened during the research process.

## Guidelines and Standards Statement

No standardised guidelines are available for the research method applied in this study.
